# Melioidosis in goats at a single Australian farm was caused by multiple diverse lineages of *Burkholderia pseudomallei* present in soil

**DOI:** 10.1371/journal.pntd.0012683

**Published:** 2024-12-19

**Authors:** Joseph D. Busch, Mirjam Kaestli, Mark Mayo, Chandler C. Roe, Adam J. Vazquez, Jodie Low Choy, Glenda Harrington, Suresh Benedict, Nathan E. Stone, Christopher J. Allender, Richard A. Bowen, Paul Keim, Bart J. Currie, Jason W. Sahl, Apichai Tuanyok, David M. Wagner

**Affiliations:** 1 Pathogen and Microbiome Institute, Northern Arizona University, Flagstaff, Arizona, United States of America; 2 Menzies School of Health Research, Charles Darwin University, Casuarina, Northern Territory, Australia; 3 Department of Industry, Tourism and Trade, Berrimah Veterinary Laboratory, Berrimah, Northern Territory, Australia; 4 Department of Biological Sciences, Colorado State University, Ft. Collins, Colorado, United States of America; 5 Department of Infectious Diseases and Immunology, College of Veterinary Medicine, and Emerging Pathogens Institute, University of Florida, Gainesville, Florida, United States of America; Institut Pasteur, FRANCE

## Abstract

**Background:**

*Burkholderia pseudomallei*, causative agent of melioidosis, is a One Health concern as it is acquired directly from soil and water and causes disease in humans and agricultural and wild animals. We examined *B*. *pseudomallei* in soil and goats at a single farm in the Northern Territory of Australia where >30 goats acquired melioidosis over nine years.

**Methodology/Principal findings:**

We cultured 45 *B*. *pseudomallei* isolates from 35 goats and sampled soil in and around goat enclosures to isolate and detect *B*. *pseudomallei* and evaluate characteristics associated with its occurrence; 33 soil isolates were obtained from 1993–1994 and 116 in 2006. Ninety-two goat and soil isolates were sequenced; mice were challenged with six soil isolates to evaluate virulence. Sampling depth and total N/organic C correlated with *B*. *pseudomallei* presence. Twelve sequence types (STs) were identified. Most goat infections (74%) were ST617, some with high similarity to 2006 soil isolates, suggesting ST617 was successful at persisting in soil and infecting goats. ST260 and ST266 isolates were highly virulent in mice but other isolates produced low/intermediate virulence; three of these were ST326 isolates, the most common soil ST in 2006. Thus, virulent and non-virulent lineages can co-occur locally. Three genes associated with virulence were present in ST260 and ST266, absent in most ST326 isolates, and present or variably present in ST617.

**Conclusions/Significance:**

Agricultural animals can influence *B*. *pseudomallei* abundance and diversity in local environments. This effect may persist, as *B*. *pseudomallei* was detected more often from soil collected inside and adjacent to goat enclosures years after most goats were removed. Following goat removal, the low virulence ST326, which was not isolated from soil when goats were present, became the predominant ST in soil by 2006. Although multiple diverse lineages of *B*. *pseudomallei* may exist in a given location, some may infect mammals more efficiently than others.

## Author summary

We studied genomic diversity in the environmental bacterial pathogen, *Burkholderia pseudomallei*, which caused the disease melioidosis in goats at a small farm in northern Australia. By comparing genomes from 92 *B*. *pseudomallei* isolates from goats and soil, we discovered 12 diverse lineages at this location, three of which infected the majority of goats and were cultured from the soil of goat enclosures. We also conducted a mouse challenge experiment using six isolates from this farm and determined that two soil isolates displayed much greater virulence than the others. Finally, we investigated soil factors associated with *B*. *pseudomallei* occurrence and found 1) most *B*. *pseudomallei* isolates were sampled from irrigated soils, and 2) *B*. *pseudomallei* was much more likely to be encountered at soil depths of 30 cm than 10 cm. The presence of this pathogen in the soil severely impacted maintenance of the goat herd due to high mortality and emphasizes its One Health implications for other livestock operations in tropical countries where *B*. *pseudomallei* is endemic. Our findings provide insight into the importance of more fully understanding soil habitats that promote *B*. *pseudomallei* presence, thereby increasing the risk of melioidosis to humans and agricultural animals.

## Introduction

*Burkholderia pseudomallei* is a pathogen of One Health importance that occurs in soil and water, is endemic to Southeast Asia and northern Australia, and is increasingly reported from other regions of the world [1,2]. This bacterium is the causative agent of melioidosis, a potentially fatal disease in humans [3]. Nearly all human cases are acquired directly from the environment via contact with soil or unchlorinated water [4]. This free-living saprophyte has increasingly been discovered in the environment in other regions of the world, including Africa, the Caribbean, and the Americas [2,5–8]. In humans, melioidosis is typically an opportunistic infection of patients with particular risk factors, such as diabetes or immunocompromised condition [9]. Thailand has by far the greatest number (2,000–3,000) of reported human melioidosis cases each year, where *B*. *pseudomallei* causes 19% of community-acquired bacteremia and agricultural workers are among the highest at-risk population to develop infection due to repeated exposure to *B*. *pseudomallei*, primarily due to direct contact with compromised skin [10]. In tropical northern Australia, melioidosis was historically the most common cause of fatal bacteremia pneumonia in humans [11,12], but with improved diagnosis and therapy, mortality from melioidosis has now decreased to under 10% [13].

Melioidosis has been described in over 50 species of animals [9,14]. Although animals native to endemic regions can be infected by *B*. *pseudomallei*, many are thought to be resistant [15]. In contrast, many agricultural and exotic animals imported into endemic regions are highly susceptible, especially sheep [16] and camelids [17,18]. Goats are also highly susceptible [19] and exhibit diverse presentations [20], whereas pigs are less susceptible and can even have asymptomatic internal infections [21]. In Thailand, the incidence of melioidosis in goats is higher in the wet season and generally parallels the incidence of human cases within each province, with the lowest number of cases in the central and southern regions of the country [22]. Animal melioidosis has also been described in livestock (goats, sheep, and others) from southern China [23] and Iran [24]; however, the true impact of this disease in animals remains unknown in many countries.

In Australia, animal melioidosis was first described in sheep from Queensland in 1949 [16], which is also the state where the first human case was described in Townsville in 1950 [25]. Most human and animal melioidosis cases occur during the wet season in tropical northern Australia [19], where *B*. *pseudomallei* is recognized as a significant One Health concern due to morbidity and mortality in livestock and its ability to contaminate unpasteurized milk [26,27]. Small melioidosis outbreaks in agricultural animals have been reported in Australia [28], and goats and sheep are considered to be especially susceptible to the disease [19,29]; experimental work supports transplacental infection as one route of transmission in goats [30]. Melioidosis can also rarely occur in native animals in tropical Australia, such as an outbreak described in juvenile saltwater crocodiles from contamination of wild-harvested eggs during farm incubation [31]. Melioidosis is also well recognized in imported exotic animals in zoos in endemic locations, as in a recent cluster of slender-tailed meerkats in a Darwin wildlife park [32].

Human activities that disturb soil, such as agriculture, the introduction of livestock, irrigation, gardening, and infrastructural developments are known to positively influence the occurrence and abundance of *B*. *pseudomallei* in Australia, as well as soil factors including lower soil pH, soils with imperfect drainage, and the presence of oxidized iron such as redoxic hydrosols with a ferricrete layer [33–35]. As awareness of this pathogen grows it has become more widely accepted that *B*. *pseudomallei* is an emerging One Health pathogen that is incompletely understood. Nevertheless, it is important when including *B*. *pseudomallei* as a One Health pathogen to emphasize that it is not a zoonosis, with melioidosis in humans and animals each occurring independently via acquisition from the environment and almost never via transmission between animals and humans [9].

*Burkholderia pseudomallei* exhibits high levels of genetic recombination [36,37] and a large accessory genome [38]. Phylogenetic reconstruction based on whole genome sequences have revealed extensive phylogenetic diversity in Australia and Asia, with the most basal and diverse isolates originating from Australia [37,38]. Isolates from diverse multilocus sequence types (STs) have been found to cause disease [39], with several responsible for the majority of human infections in both Thailand (ST54, ST58, ST70, ST167) [40] and Australia (ST109, ST132, ST259, ST283, ST434) [41]. Over 550 putative virulence loci have been described in *B*. *pseudomallei* [42–46], but not all loci are required for a successful infection and virulence appears to be associated with loci that are variably present across isolates [47,48]. Identification of the specific combinations of different virulence loci that facilitate infections in humans remains a critically important knowledge gap for this pathogen.

The setting for this study was a rural goat farm near Darwin in the Northern Territory of Australia where melioidosis affected a large proportion of goats across multiple years (1992–2001) [19,49,50]. An isolate cultured from a goat on this farm (MSHR511; ST617) has been shown to be lethal in an experimental caprine infection model [51,52]. Melioidosis became a significant challenge for goat production at this farm, to the point that all goats either were euthanized, died of the disease, or were removed from the premises. In this study, we describe a comprehensive investigation of the soil environment and genomically characterize *B*. *pseudomallei* isolates obtained from both goats and soil at this farm to address the following questions: 1) What abiotic factors were associated with the presence of *B*. *pseudomallei* in soil at this site? 2) What level of phylogenetic diversity existed among *B*. *pseudomallei* isolates collected from soil at a small spatial scale? 3) What subset of the phylogenetic diversity of *B*. *pseudomallei* present in the soil was also found in goats? 4) Which lineages of *B*. *pseudomallei* infected the largest number of goats? We used whole genome sequencing to characterize 92 *B*. *pseudomallei* isolates from this site (23 from goats and 69 from soil) and chose six isolates for an experimental mouse challenge study that identified differences in virulence across multiple *B*. *pseudomallei* lineages.

## Methods

### Ethics statement

Mouse challenge experiments were approved by the Animal Care and Use Committee of Colorado State University (CSU), protocol number 17-7497A. The experiments were performed under Select Agent and ABSL-3 containment practices at CSU and in strict accordance with the recommendations in the Guide for the Care and Use of Laboratory Animals of the National Institutes of Health. Every effort was made to minimize animal suffering and pain.

### Sample collection and culturing

#### Overview

**Fig 1:**
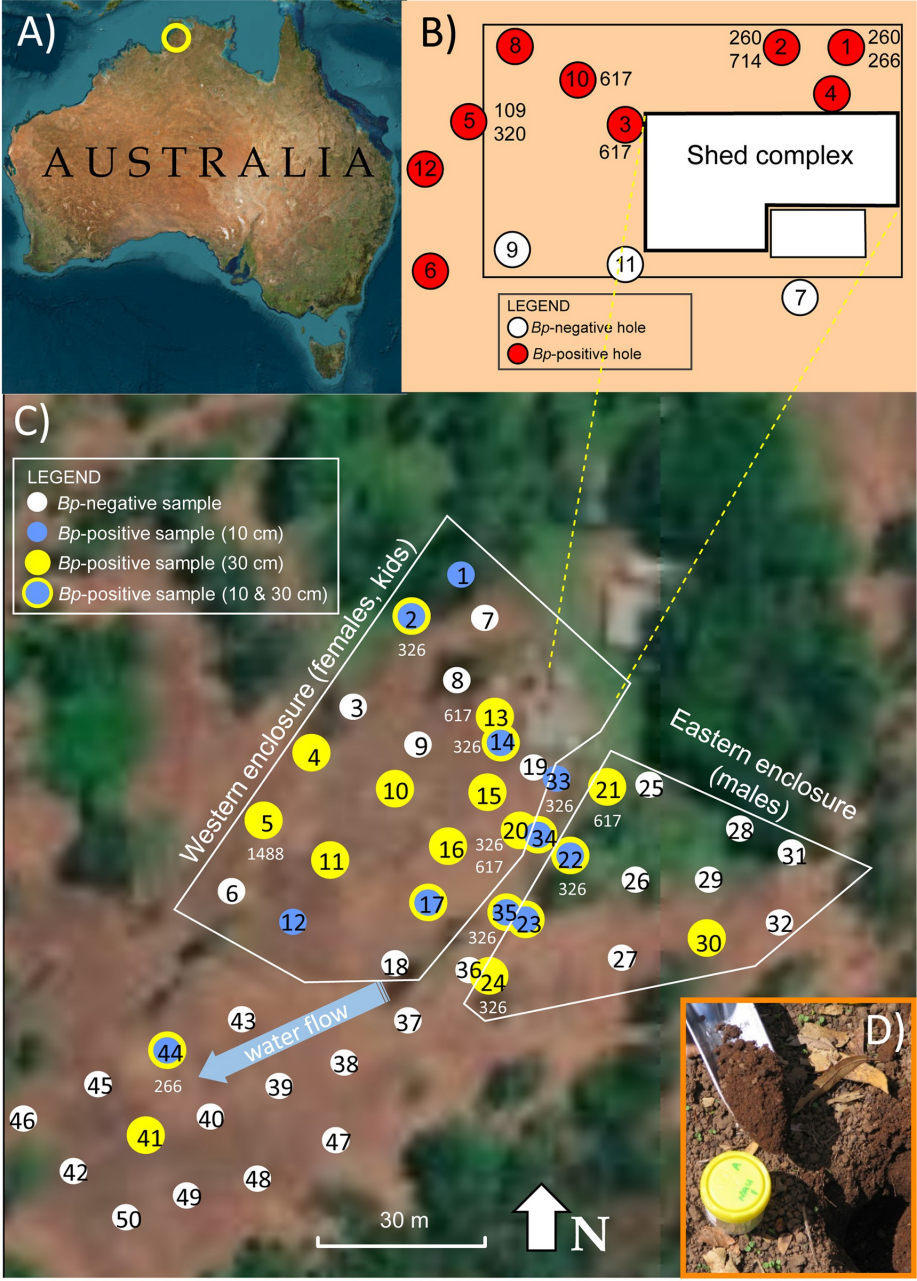
Locations where soil samples were collected and tested for the presence of *Burkholderia pseudomallei* at a rural farm south of Darwin, Australia. **Panel A)** Location of study site in northern Australia. **Panel B)** Soil study #1 was conducted between March 1993 –March 1994 by repeatedly sampling 12 holes surrounding a shed complex in the western goat enclosure. Red circles show holes from which *B*. *pseudomallei* was cultured; white circles were culture negative. Soil samples were collected at 1–5 depths per hole (depths ranging from 10 cm to 90 cm). Numbers adjacent to five of the nine positive holes denote the STs of cultured isolates; MLST data were not generated for the remaining four positive holes. **Panel C)** Soil study #2 was conducted in November 2006 by sampling 50 holes (numbered circles) in a systematic grid with two soil samples collected per hole (10 and 30 cm depths). The presence of *B*. *pseudomallei* was determined either by culturing or real-time PCR (see Methods). Numbers adjacent to positive holes (yellow = 30 cm; blue = 10 cm) denote the STs of cultured isolates; MLST data were not generated for every positive hole. The direction of water drainage at this site is noted with a blue arrow. **Panel D)** 2006 soil sample 1A (10 cm depth) was found to be positive for *B*. *pseudomallei* using real-time PCR; photograph by David Wagner. The maps in panels A and C (World Imagery) were created using ArcGIS software by Esri. ArcGIS and ArcMap are the intellectual property of Esri and are used herein under license. Copyright Esri. All rights reserved. For more information about Esri software, please visit www.esri.com. Basemap for panel A and C: World Imagery (WGS84), https://usgs.maps.arcgis.com/home/item.html?id=10df2279f9684e4a9f6a7f08febac2a9.

This study was initiated in response to frequent cases of melioidosis in goats at a rural farm near Darwin in Northern Territory, Australia (**Fig 1A**), that began in 1992 [19,49,50]. With the owner’s permission, we repeatedly visited the study site to sample goats and soil to culture *B*. *pseudomallei*. We addressed four study questions (listed above) by generating three main datasets: 1) genomes of *B*. *pseudomallei* isolates that originated from goats (collected in 1992–2001) and soil samples (collected in 1992–93 and 2006), 2) soil chemistry analysis from 2006 samples, and 3) mortality of mice experimentally challenged with six *B*. *pseudomallei* isolates cultured from soil.

#### Goat isolates

Up to 30 goats were kept at this farm at any one time in two main enclosures, one for females and kids and the other for males. The Menzies School of Health Research (Menzies) began sampling *B*. *pseudomallei* from goats at this study site in 1992. Animals presented with a range of symptoms, including lethargy, skin lesions, persistent cough, fever, and other symptoms of severe infection. To attempt culturing of *B*. *pseudomallei*, noninvasive samples, including milk, throat and mucus swabs, swabs from skin lesions (ear and udder), and feces, were taken from animals with less severe symptoms. Animals with severe illness were euthanized and organs (lungs, aorta, liver, kidney, spleen, and lymph nodes) were harvested for culturing. Samples of these tissues were also submitted for pathology analysis at Berrimah Veterinary Laboratories (Department of Industry, Tourism and Trade, Berrimah, NT, Australia). Animal samples were inoculated onto tryptic soy agar with sheep’s blood, MacConkey agar, and Ashdown’s selective agar [53] (Oxoid Australia Pty Ltd, Thebarton, South Australia 5031) and incubated at 35°C for 48 hours. Concurrently, the samples were also inoculated into Ashdown’s broth and incubated at 35°C for seven days. If *B*. *pseudomallei* was not observed on the agar plates, the Day 7 incubation of Ashdown’s broth was subcultured onto Ashdown’s agar and incubated at 35°C for another 48 hours. The isolates were identified to species level using a standardized biochemical assay panel for Enterobacteriaceae, API 20 NE (bioMérieux, Marcy-I’Etoile, France), and stored in cryogenic broth with glycerol for referral to Menzies laboratory.

A total of 195 *B*. *pseudomallei* isolates were collected in this study for further characterization (**S1 Table**). These isolates originated from three sources: 1) 45 isolates cultured from 35 goats between 1992–2001, 2) 33 isolates cultured from soil study #1 (March 1993-March 1994) that focused on a shed complex in the western enclosure (**Fig 1B**), and 3) 116 isolates cultured from soil study #2 in November 2006 (**Fig 1C**). We included one additional goat isolate (MSHR0199) that was collected on 01April 1993 at a separate farm 25 km distant, bringing the total number of goat isolates to 46. The two soil studies were conducted to investigate possible associations between *B*. *pseudomallei* in goats and soil, since nearly all hosts acquire this pathogen directly from the environment [9].

#### Soil study #1

This first soil study was focused on detecting *B*. *pseudomallei* at a heavily used goat shed complex during wet versus dry seasons. The sheds provided food, water, and shade for female and kid goats (**Fig 1B**) and were expected to be the most likely place to detect and culture *B*. *pseudomallei* from disturbed soil. A total of 128 soil samples were collected over four sampling sessions (24 March 1993, 17 November 1993, 15 December 1993, and 28 March 1994) that spanned 13 months (**S2 Table**). Approximately 100 g of soil were collected for each sample. The soil in this area is classified as red Kandosol, a common soil type in the wider Darwin region [33]. Soil samples were collected from 12 holes, typically at 3–4 different depths per hole in the range of 0–90 cm, but in some sessions certain holes were sampled at only one depth. Eleven holes were sampled multiple times, and five (holes 1, 2, 4, 5, and 6) were sampled during all four sessions.

The 128 samples from soil study #1 were the source of 33 *B*. *pseudomallei* isolates cultured at the Menzies laboratory using Ashdown’s selective media [53] and a modified environmental culturing procedure [54]. The procedure started by suspending 20 g of soil in 20 mL of sterile distilled water and incubating at 37°C while shaking (250 rpm) with air availability for 48 hours. Samples were allowed to settle for 1 hour and then 100 μL of the water suspension was plated onto Ashdown’s agar plates. Also, 10 mL of water suspension was inoculated into 10 mL of Ashdown’s broth (containing 0.05 mg/mL colistin instead of gentamycin); the Ashdown’s broth was then shaken at 37°C for seven days. We plated 10 μL of the top layer from the Ashdown’s broth suspension onto Ashdown’s agar plates (containing 4 mg/mL gentamycin) at Day 2 and Day 7 post broth inoculation. After incubating agar plates for 48 hours at 37°C, we sub-cultured single colonies onto chocolate agar. Cultures were streaked from a single colony to form a lawn and then stored as -80°C glycerol stocks in Luria-Bertani (LB) broth with 20% glycerol. One *B*. *pseudomallei* isolate derived from a single colony was saved in a permanent frozen glycerol stock at Menzies to represent each individual soil sample.

#### Soil study #2

The goal of the 2006 soil study was to systematically collect soil across a larger grid that included holes located inside and outside of the two goat enclosures to infer associations among *B*. *pseudomallei* presence, previous presence of goats, and soil chemistry variables. Soil study #2 was conducted as a single sampling session that took place 07–08 November 2006 at the end of the dry season in northern Australia. All goats had been removed from this farm prior to 2004. By 2006, two goats were again present; both were healthy and exhibited no signs of melioidosis at the time of sampling. We first set up a grid of 50 sampling points spaced 3–15 meters apart (**Fig 1C**) using handheld Trimble GPS units (Westminster, CO, USA). The sampling grid purposefully excluded the shed complex of the western enclosure (due to presence of *B*. *pseudomallei* in soil study #1) and extended across two notably different habitats: 1) an irrigated northern area (holes 1–36) with vegetation cover in the two goat enclosures and a grassy walkway between them, and 2) a dry, bare southern area (holes 37–50) slightly downslope of the goat enclosures. At each sampling point we collected ~100 g of soil (**Fig 1D**) at two depths per hole: 10 cm (designated sample A) and 30 cm (sample B) for a total of 100 samples (**S2 Table**). Our culturing procedure followed consensus guidelines [55,56] for environmental surveys of *B*. *pseudomallei* as described above for soil study #1. However, in soil study #2 we attempted to sub-culture a greater number of colonies onto chocolate agar to investigate phylogenetic diversity within and among individual soil samples. Therefore, up to 16 single colonies per soil sample were selected for permanent storage in the form of -80°C glycerol stocks in LB broth with 20% glycerol (**S2 Table**). All *B*. *pseudomallei* isolates from 2006 (n = 116) were initially cultured at the Menzies laboratory, and then live samples of each were shipped to Northern Arizona University (NAU) under Select Agent compliant procedures to perform additional phylogenetic and genomic analyses.

### Soil analysis–soil study #2

A portion of each soil sample from soil study #2 was sent to a commercial lab (CSBP Limited, Kwiwana Beach, WA, Australia) to measure nine physicochemical soil variables that we treated as either continuous numeric variables (total N, organic C estimated by loss on ignition [LOI], conductivity, pH in H_2_O, pH in CaCl_2_, moisture, and gravel %), or categorical variables (soil texture and color) in the statistical analysis; a detailed description of each variable is provided in **S2 Table**. We also calculated the ratio of total N/organic C. The choice of abiotic factors to test was based on their availability, our knowledge of Australian soils at the time of the study, and subsequent analysis [57–59]. Any multi-category variables were simplified into binary variables to avoid model overparameterization and scarce data for some categories. For example, soil texture was sorted into the most abundant level (clay) versus all other textures, and soil color as red/orange versus all other colors. In addition to the nine soil variables listed above, we recorded five site factors for each hole and soil sample: depth of soil sample (10 vs. 30 cm), hole location in relation to goat enclosures (inside vs. outside; *i*.*e*., holes 1–32 vs. 33–50), hole location in relation to goat sheds (inside shed vs. outside), vegetation at the hole (presence/absence), and location within two main grid areas: northern (holes 1–36) vs. southern (holes 37–50) (**Fig 1C** and **S2 Table**). These five site factors were treated as binary variables in the statistical analyses described below. All abiotic soil variables and site factors were used to build a predictive model for the presence of *B*. *pseudomallei* at this small spatial scale [59].

The presence of *B*. *pseudomallei* in soil was determined for all soil samples by culturing as described above, as well as screening soil DNA extractions with a real-time PCR assay [60] that targets *orf2* in the type three secretion system 1 (TTS1) cluster within the genome of *B*. *pseudomallei*. This target is highly specific to *B*. *pseudomallei* [38] and is considered the gold standard for PCR-based detection of this pathogen. MoBio PowerSoil extraction kits (Carlsbad, CA, USA) were used to extract DNA from 500 mg of soil according to manufacturer’s instructions. All soil DNA extracts were screened with the TTS1 assay on an ABI 7900 machine (Applied Biosystems, Foster City, CA, USA). We used DNA from *B*. *pseudomallei* K96243 as our positive control and molecular grade water for all no-template controls (NTCs). Soil samples were deemed positive for *B*. *pseudomallei* if they were culture positive and/or positive by real-time PCR.

### Statistical analysis of soil study #2

Statistical analyses were conducted in R v4.1.3 (R Project for Statistical Computing, Vienna, Austria). The association between *B*. *pseudomallei* occurrence and abiotic factors in soil study #2 was explored with uni- and multivariable binomial generalized linear mixed models (GLMMs), in which abiotic soil variables and site factors were used as predictors and the binary outcomes were defined as *B*. *pseudomallei* occurrence or absence; binomial generalized linear models are also known as logistic regressions. All models (univariable and multivariable) accounted for soil depth at 10 vs. 30 cm (as a binary predictor), and spatial autocorrelation both within holes (by using holes as a random effect, *i*.*e*. random intercept [61]) and among holes (using a spatial exponential covariance structure). With 100 soil samples, there was an 81% probability to get 20 positive samples allowing moderate statistical power to fit a simple multivariable model with three parameter estimates. This was with an expected intra-hole correlation of 0.3 (effective sample size 77) and a *B*. *pseudomallei* detection rate of 30% (chosen because the detection rate in soil study #1 was 26%). Models were fitted using Penalized Quasi-Likelihood (glmmPQL) of the “MASS” package in R, which allows fitting of the spatial covariance structure [62,63]. Two variables (pH in CaCl_2_ and shed) were removed from the final analysis; pH in CaCl_2_ was excluded due to collinearity with pH in H_2_O and shed was excluded because only 8 samples (in 4 holes) were collected inside sheds. The other 13 soil variables and site factors from the above list were included. Model residuals were checked for lack of spatial autocorrelation (variograms with package geoR) and lack of patterns across fitted values and predictors (package DHARMa). Models were also checked for no collinearity using the variance inflation factor; bare soil was excluded due to strong collinearity with location. Each numerical and binary predictor was tested in a univariable model (with covariate depth and random effect hole); a multivariable model was fitted using model averaging with the package MuMIn specifying a maximum of four predictors in a model and using AICc as a model selection tool; top models with delta AICc <2 were averaged.

We further explored fine-scale patterns of the abiotic soil variables within the context of two site factors that were associated with *B*. *pseudomallei* occurrence in the above GLMMs: 1) location within the two sampling grid areas (northern vs. southern) and 2) soil depth (10 cm vs. 30 cm). Differences in abiotic factors between samples were assessed with GLMMs (Gaussian, binomial or Gamma with log link) accounting for spatial autocorrelation and depth (when appropriate) as described above. Spatial autocorrelation across holes for *B*. *pseudomallei* occurrence was assessed by plotting the semivariance and Moran’s I correlogram with P values based on permutations (library EcoGenetics). Longitude/latitude coordinates were converted to WGS84 UTM zone 52S projected data (library rgdal). All tests were two-tailed and considered significant for P-values < 0.050.

### Genome sequencing and phylogenetic analysis

#### DNA extraction

To extract DNA for genome sequencing, individual isolates of *B*. *pseudomallei* were grown on Luria-Bertani agar (LBA) and incubated at 37°C for 24–48 hours to extract high molecular weight DNA using the Qiagen DNeasy Blood and Tissue Kit (catalog no. 69504; Valencia, CA, USA). We followed the Gram-positive protocol with the addition of 1 mg/mL of lysozyme and doubled all volumes.

#### Genome sequencing and assembly

We sequenced 92 *B*. *pseudomallei* genomes, including isolates from goat (n = 23), soil study #1 (n = 7), and soil study #2 (n = 62) (**S3 Table**). Using approximately 2.7 μg of gDNA, libraries were prepared for whole genome sequencing as previously described [64]. Most genomes were sequenced on Illumina short-read platforms, a 2x100 bp HiSeq run, or a 2x250 bp MiSeq run, but a few isolates were sequenced using a GAIIx sequencer.

Sequence reads were assembled with the SPAdes assembler v.3.10.0 [65]. The per contig coverage was determined by mapping reads against contigs with Minimap2 v2.17 [66] and calculating coverage with Samtools v1.9 [67]. Each genome assembly was manually edited to remove contigs that had an anomalously low coverage compared to the rest of the assembly or aligned against known contaminants based on a BLASTN alignment [68] against the GenBank nt database [69]. Representative genome assemblies were submitted to GenBank and assembly statistics and accession numbers are shown in **S3 Table**.

#### *In silico* genotyping

The multi-locus sequence type (MLST) for each isolate (**S1 Table**) was taken from its genome sequence with fastmlst v0.0.15 [70]. Nine of the isolates not chosen for WGS had been independently sequenced at seven MLST loci by Menzies and uploaded to the pubMLST database for *B*. *pseudomallei* (https://pubmlst.org/organisms/burkholderia-pseudomallei), including MSHR0124, MSHR0212, MSHR0215, MSHR0219, MSHR0231, MSHR0294, MSHR0298, MSHR0408, and MSHR0484. Four of these isolates carried STs (ST109, ST131, ST320, and ST714) that were not sequenced with the 92 new genomes. We also queried the *B*. *pseudomallei* pubMLST database on 22 February 2024 for all instances of any ST that we identified. Starting at the *B*. *pseudomallei* pubMLST website (above), we chose the “Typing” icon; under “Search for allelic profiles” we used the “By specific criteria” option. On the query page, we changed the Scheme field to “MLST”; in the Locus/scheme fields we selected “ST =“ and added the ST number; in the Display/sort options fields we selected ST, ascending, and display all. We then selected the ST icon for each record returned; this directs the user to a new page and under Client database we selected the icon for “# isolates”. The final page provides a list of all records available for each ST with metadata that can be exported.

#### Comparative pan-genomics

All genome assemblies were annotated with Prokka v1.14.6 [71]. The pan- and core-genomes were calculated for each ST with Panaroo v1.2.3 [72]. The resulting pan-genome representative sequences were mapped against genome assemblies with the large-scale BLAST score ratio (LS-BSR) tool v1.2.3 [73] in conjunction with BLAT v36x2 [74]. Variably-conserved genes, based on blast score ratio (BSR) values [75], were mapped against phylogenies from each ST with LS-BSR/BLAT and visualized with the interactive tree of life (iTOL) [76].

#### Single nucleotide polymorphism (SNP) identification and phylogenetics

**Fig 2:**
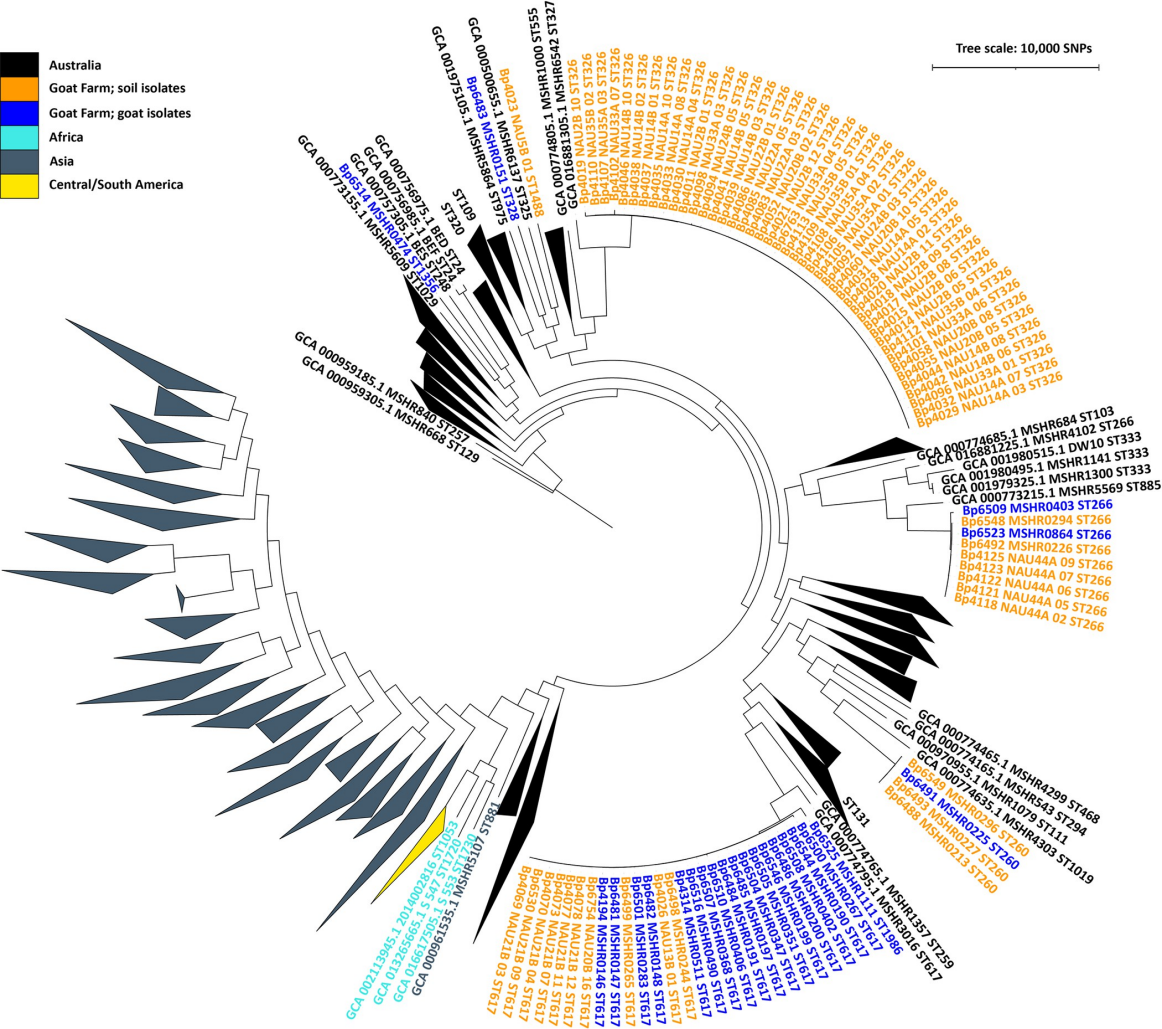
Position of goat farm isolates in a global *B*. *pseudomallei* phylogeny. This maximum parsimony phylogeny was constructed using 187,486 single-nucleotide polymorphisms (SNPs) discovered among whole genome sequences from 749 *B*. *pseudomallei* isolates obtained from the goat farm and other global locations (**S3 Table**); it is rooted on Australian isolate MSHR668. Colors of collapsed clades and individual isolates correspond to geographic origin, as indicated in the legend. ST names in text next to collapsed clades or individual reference genomes indicate the phylogenetic position of four STs (ST109, ST131, ST320, ST714) that we detected at the goat farm but did not sequence (**S1 Table**); the representatives of these STs included in this phylogeny were obtained from GenBank (**S3 Table**).

To understand the placement of 92 new genomes within the global *B*. *pseudomallei* phylogeny (**Fig 2**), genome assemblies were combined with a global set of 693 *B*. *pseudomallei* genomes (**S3 Table**); genomes were downloaded using the ncbi-genome-download tool (https://github.com/kblin/ncbi-genome-download) and dereplicated with the assembly dereplicator tool (https://github.com/rrwick/Assembly-Dereplicator) at a threshold of 0.0005. SNPs were called with nucmer v3.1 [77] in conjunction with NASP v1.2.0 [78] for genome assemblies using *B*. *pseudomallei* K96243 (accession number GCA_000011545.1) [42] as the reference. We removed 36 genomes that were either replicated or had poor breadth of coverage; the remaining 749 genomes covered 48% of the reference genome K96243. A concatenated alignment of 187,486 SNPs was used to infer a global phylogeny using maximum parsimony MEGAX [79] with the subtree-pruning-regrafting (SPR) search method. The phylogeny was rooted on isolate MSHR668 (GCA_000959305.1).

For reconstructing a separate maximum parsimony phylogeny within each ST group of isolates (*e*.*g*., ST260, ST266, ST326, and ST617), raw reads were aligned against an internal high-quality genome reference (chosen for large assembly size and low number of contigs) within each specific ST groups using minimap2 v2.24 [66], and SNPs were then called using the HaplotypeCaller method in GATK v4.1.2 [80,81]. SNPs were filtered if the coverage was <10X or the minimum allele frequency ratio was <0.90. All remaining polymorphic positions were concatenated into a single multi-FASTA file and used to infer a maximum parsimony phylogeny using MEGAX [79]. The retention index (RI) of SNP alignments, which provides information on the extent of homoplasy, was calculated with Phangorn v2.4 [82].

### *In vivo* animal challenge

#### Diverse soil isolates

To investigate the potential virulence of six soil isolates from four diverse STs (ST260, ST266, ST326, and ST617), we conducted mouse challenge experiments using BALB/c mice, a highly susceptible animal model for studying *B*. *pseudomallei* infection [83]. The six strains were MSHR0296 (ST260), NAU44-A6 (ST266), NAU14-B6, NAU24-B3, and NAU33-A4 (all ST326), and NAU21-B9 (ST617) (denoted in **S1 Table**). A set of BALB/c mice also were challenged with control strains *B*. *pseudomallei* NCTC13178 (high virulence) and *B*. *pseudomallei* NCTC13179 (low virulence) purchased from the National Collection of Type Culture (NCTC) in the UK. We used three infection routes to identify differential virulence: subcutaneous (SC), intraperitoneal (IP), and intranasal (IN). To look at differences across animal models, we also tested C57BL/6J mice with four of the six isolates using the SC and IN routes.

#### Mouse inoculations

Female BALB/c and C57BL/6J mice, 6–8 weeks old, were purchased from Charles River laboratories and housed at Colorado State University. Each *B*. *pseudomallei* isolate was grown at 37°C in Brain-heart infusion (BHI) broth medium to an OD_600_ of 1, supplemented with glycerol to 10% v/v, and frozen in multiple aliquots at -80°C. A vial of these stocks was thawed, and serial dilutions plated on BHI agar plates to determine titer two days prior to the animal challenge. On the day of the animal challenge, a new frozen aliquot was thawed and diluted with PBS to obtain approximately 500 CFU per 50 μL of the bacterial inoculum.

The LD_50_ for BALB/c mice infected via the SC route was previously reported as 1000 CFU [83]; this guided the use of 0.5x LD_50_ in this study to identify differential virulence. Each mouse (5 mice per *B*. *pseudomallei* isolate, following [84]) was subcutaneously inoculated in the medial right leg with 50 μL of the bacterial inoculum (10 CFU/μL); subcutaneous infection in mouse models represents percutaneous human infections, which are common in endemic areas [85]. For IP infection, we used 100 μL of the bacterial inoculum (10 CFU/μL) to deliver 1,000 CFU into the peritoneal cavity. For IN infection, we used 25 μL of a more concentrated bacterial inoculum (20 CFU/μL) to deliver 250 CFU into the right nares. The same volume delivered in each infection route was also back-titrated to confirm the given dose of each isolate.

After inoculation, mice were weighed and evaluated for clinical symptoms associated with *B*. *pseudomallei* for 21 days post infection. The moribund and surviving mice at 21 days post infection were euthanized by a standard CO_2_ overdosing method. Survival curves for each isolate were graphed using JMP Software (Cary, North Carolina, USA). To test the null hypothesis that the risk of death is the same for each *B*. *pseudomallei* isolate, we performed a Kaplan-Meier analysis of survival times using the nonparametric generalized Wilcoxon test.

### Analysis of virulence loci

We screened 552 previously characterized virulence factors, which are listed in S3 Table in Sahl *et al*. 2022 [86], against all six mouse challenge isolates using LS-BSR/BLAT to identify gene presence/absence that could potentially be related to virulence. CDSs were identified that were more highly conserved in the highly virulent isolates (killed all mice) compared to all others (low to moderate virulence in mice) with the compare_BSR.py script in the LS-BSR repository based on BSR values. The differential conservation of genes was verified through short read mapping approaches wherein the breadth of coverage was calculated with Samtools.

## Results

### Presence of *B*. *pseudomallei* in soil

In soil study #1, we successfully cultured *B*. *pseudomallei* from soil samples collected during each of the four sampling sessions from March 1993-March 1994 (**S2 Table**), which suggests the presence of goats and/or disturbed soil at the shed complex created conditions conducive to *B*. *pseudomallei* throughout the year. In total, *B*. *pseudomallei* was cultured from nine of 12 holes (75%; **Fig 1B**). No single hole yielded *B*. *pseudomallei* isolates in all four sessions, but holes 1, 2, and 5 yielded isolates in three sessions. Two holes (6 and 12) yielded *B*. *pseudomallei* isolates collected in two sessions, and four holes (3, 4, 8, and 10) yielded isolates in just one session. Holes 7 and 9 from 17 November 1993 and hole 11 from 15 December 1993 did not yield any *B*. *pseudomallei* isolates. Of the 128 total soil samples that we collected in soil study #1 from the 12 holes, 33 (26%) yielded *B*. *pseudomallei* isolates. The depth from which positive samples were collected ranged from 0–90 cm below the surface, but 54% of the isolates (18 of 33) were obtained from soil samples collected at depths of 20–40 cm (**S2 Table**). Concurrently, many goats had contracted melioidosis during the course of soil study #1, leading to 22 new *B*. *pseudomallei* isolates obtained from goats between March 1993 and March 1994 (**S1 Table**).

In soil study #2, we successfully detected *B*. *pseudomallei* across a systematic grid of 50 holes that were spread across a wider area of the farm. Importantly, we sampled both inside and outside the goat enclosures (**Fig 1C**). The combined use of culturing and real-time PCR detected *B*. *pseudomallei* in samples collected from 23 of 50 holes (44%; **S2 Table**). We found ten times the number of *B*. *pseudomallei*-positive holes in the northern area of the grid (n = 21) compared to the southern area with no enclosures or irrigation (n = 2), and the goat enclosures yielded >3 times as many *B*. *pseudomallei*-positive holes as those outside the enclosures (18 vs. 5). The western enclosure for females and kids contained 56% (13 of 23) of the total *B*. *pseudomallei*-positive holes. We more frequently detected *B*. *pseudomallei* at just a single depth in a given hole rather than at both depths (16 vs. 7). Regarding the 100 soil samples collected in 2006, 31 (31%) were positive for *B*. *pseudomallei* and we cultured a total of 116 *B*. *pseudomallei* isolates from these 31 samples (**S1 Table**). Interestingly, successful detection of *B*. *pseudomallei* by both methods (*i*.*e*., culturing and TTS1 PCR) from individual 2006 soil samples occurred only ~50% of the time (15 of 31 positive samples).

### Analysis of soil variables and abiotic factors

The results of analyses of associations between *B*. *pseudomallei* occurrence and soil variables/site factors based upon uni- and multivariable binomial GLMMs are shown in **Table 1**. In the univariable analyses (upper portion of **Table 1**), the ratio of total N to organic C was significantly different between *B*. *pseudomallei* positive and negative soil samples, with higher ratio values being more frequently associated with *B*. *pseudomallei* occurrence (P = 0.016). The pH in H_2_O was slightly less acidic in *B*. *pseudomallei* positive vs. negative soil samples, but the difference was not significant (P = 0.088). When considering binary site factors (lower portion of **Table 1**), sample depth was important, with almost twice as many soil samples positive at 30 cm (40%, n = 20/50) as at 10 cm (22%, n = 11/50; P = 0.001). Location within the sampling grid (*i*.*e*., grid area–north or south) was also important, as the odds of detecting *B*. *pseudomallei* in the irrigated northern area were 6.7 times higher compared to the southern area without irrigation (P = 0.016). Due to the high density of positive soil samples in the northern area, spatial autocorrelation was observed for *B*. *pseudomallei* occurrence in holes located within 30 meters of each other (Moran’s I p<0.05). The same general pattern was found using a semivariogram plot (**S1 Fig**).

**Table 1:** Soil variables from soil study #2 (08 November 2006) were analyzed in generalized linear mixed models to evaluate associations with the presence of *B*. *pseudomallei*. OR is the odds ratio; brackets show 95% confidence intervals of OR values; P-values are shown last and * indicates P-value <0.05. The upper portion of the table reports median and min-max range values for seven numeric predictors measured in all 100 collected soil samples that were either *B*. *pseudomallei* positive (n = 31) or negative (n = 69). Conductivity values were transformed to a natural logarithm (ln) scale. The lower portion of the table shows the results for six binary predictors and reports the percentage of *B*. *pseudomallei* positive soil samples (n) out of the total soil samples (n_tot_) in that category (n/n_tot_); “ref” is the reference level category. All models (including univariable) had hole as a random effect (intra-cluster correlation 0.4) and depth as a covariate (explanatory variable). Four of the variables were fitted in a multivariable model and the conditional averaged estimates are shown (see Methods for details). Vegetation was excluded from the final multivariable model due to collinearity with grid area (probably due to irrigation of the northern area).

Soil Variable		Median (min-max range)		OR for *B*. *pseudomallei* occurrence [95% CI], P-value	
Numeric predictors	Unit	*Bps* positive samples	*Bps* negative samples	Univariable Models	Multivariable Model
Total N	%	0.12 (0.04–0.33)	0.10 (0–0.62)	11 [0.1–2,000], 0.34	
Organic C	%	6.2 (2.2–10.4)	5.5 (2.2–15.9)	1.0 [0.8–1.3], 0.95	
Ratio Total N/Organic C	x10e3	21.3 (11.7–40.5)	18.6 (0–63.4)	1.1 [1.0–1.2], 0.016*	1.1 [1.0–1.2], 0.12
Conductivity	uS/cm	46.0 (10.0–248)	36.0 (9.0–419)	1.4 [0.7–2.8], 0.29	
pH in H_2_O	-	5.8 (5.0–6.7)	5.6 (4.8–6.5)	4.6 [0.8–25.4], 0.088	3.7 [0.2–64.2], 0.37
Soil moisture	% vsw	16.0 (11.0–20.0)	15.0 (7.0–24.0)	1.0 [0.8–1.2], 0.93	
Gravel	%	0 (0–7.5)	0 (0–27.5)	1.0 [0.9–1.1], 0.92	
**Binary predictors**	**Level**	**% *Bps* positive samples (n/n**_**tot**_ **)**			
Sample depth	10 cm	22.0% (11/50)			ref
	30 cm	40.0% (20/50)			4.0 [1.8–9.1], 0.001*
Grid area	South	10.7% (3/28)			ref
	North	38.9% (28/72)			6.7 [1.4–32.6], 0.016*
Goat enclosure	No	22.2% (8/36)			ref
	Yes	35.9% (23/64)			2.2 [0.7–7.2], 0.19
Vegetation	No	17.6% (6/34)			ref
	Yes	37.9% (25/66)			4.8 [0.9–24.8], 0.062
Texture–Clay	No	35.6% (16/45)			ref
	Yes	27.3% (15/55)			0.5 [0.1–1.6], 0.22
Red/orange soil color	No	27.3% (18/66)			ref
	Yes	38.2% (13/34)			1.4 [0.3–6.2], 0.65

Of the four predictors in the multivariable analysis, only sample depth was significantly different between *B*. *pseudomallei* positive and negative soil samples (**Table 1**). Model averaging revealed that soil depth was the most important predictor for *B*. *pseudomallei* presence and it was retained in all seven top models within delta 2 AICc. The other variables, ranked in order of importance, included grid area (northern vs southern–present in 4/7 top models), the ratio of total N over organic C (4/7), vegetation (2/7), and pH (2/7). Vegetation was excluded from the multivariable model due to collinearity with grid area (likely due to irrigation).

We further explored abiotic soil variables at a fine scale within the context of the two most important site factors associated with *B*. *pseudomallei* occurrence: sample depth (10 vs. 30 cm) and grid area (irrigated northern vs. southern area). The northern area containing the goat enclosures had significantly more vegetation, total N, organic C, and a higher ratio of total N/organic C (Table A in **S4 Table**). Accordingly, the salinity was higher and the pH less acidic in the northern area. However, the northern and southern areas had similar levels of gravel and clay. A comparison of soil variables at 10 cm versus 30 cm revealed significant differences in every soil variable (Table B in **S4 Table**), even though values were highly correlated within each hole overall (Pearson >0.8, p<0.001, intra-cluster correlation 0.4). Overall, soil samples collected at 30 cm were less saline, had lower moisture, and contained less nutrients (total N, organic C, and ratio of total N/organic C) compared to soil samples collected at 10 cm. The soil samples collected at 30 cm were much more likely to be red or orange in color (68% of 30 cm samples compared to 0% at 10 cm), suggesting a greater proportion of oxidized iron in the deeper soils at this study area. Reddish-brown and reddish-grey soils have been reported as an important predictor for the presence of environmental *B*. *pseudomallei* [33]. The soil color also matches the soil classification of this area, which is a red Kandosol, which is well drained, gravelly, yellow or red earth, that is often overlaying weathered, iron-rich material [33].

### Phylogenetic diversity

A high level of phylogenetic diversity was observed among *B*. *pseudomallei* isolates obtained from goats and soil at this single farm. Within a global *B*. *pseudomallei* phylogeny (**Fig 2**) the goat farm isolates were interspersed among phylogenetic clades made up of isolates obtained from other diverse locations in Australia. Because there was a strong correlation between phylogenetic clade and ST in this phylogeny, we hereafter refer to these phylogenetic clades by ST. We note that four STs identified from the goat farm (ST109, ST131, ST320, and ST714; **Table 2**) were not sampled in the 92 new genomes and, thus, are not represented in **Fig 2** by isolates from the goat farm but are represented by reference isolates. We identified 12 STs in total from the goat farm (**Table 2**), all previously reported only from Australia, including eight that have been associated with melioidosis in humans and five previously associated with melioidosis in animals (outside of this study). Three STs were found in both goats and soil, four were found only in goats, and five were found only in soil (**Table 2**). The highest diversity was encountered in the western enclosure shed complex where all 12 STs were present in soil and/or female/kid goats that frequently used those sheds (**S1 Table**).

**Table 2:** The 12 multi-locus sequence types (STs) of *B*. *pseudomallei* found at the goat farm. The pubMLST database for *B*. *pseudomallei* [87] was used to identify the number of previous human and animal samples assigned to these STs in other studies. ST1986 has not been reported previously in pubMLST. Six soil isolates were used to evaluate virulence in mouse challenge experiments.

ST	Other cases reported in pubMLST for humans/ animals	^a^Source(s) in this study (# of isolates)	Soil isolates tested in mouse challenge	^b^Mouse model(s)	Virulence in mice
131	83/1	Goat (1)			
328	0/0	Goat (1)			
1356	1/0	Goat (1)			
1986	New ST	Goat (1)			
260	1/0	Goat (1), SS#1 (4)	MSHR0296	B, C	High
266	12/0	Goat (2), SS#1 (2), SS#2 (5)	NAU44A-06	B, C	High
617	1/0	Goat (20), SS#1 (2), SS#2 (8)	NAU21B-09	B, C	Intermediate
109	181/15	SS#1 (2)			
320	11/2	SS#1 (1)			
714	0/0	SS#1 (1)			
326	1/0	SS#2 (48)	NAU14B-06	B	Intermediate
			NAU24B-03	B, C	Low
			NAU33A-04	B	Low
1488	1/0	SS#2 (1)			

^a^ SS#1 = Soil study #1; SS#2 = Soil study #2; list of all samples/isolates is provided in **S1 Table**

^b^ B = BALB/C; C = C57BL/6J

*Burkholderia pseudomallei* isolates with genotype ST617 were recovered in goats in six consecutive years (1992–1997) and comprised 74% (20 of 27) of the goat isolates we sequenced (**S1 Table**). This ST was cultured from soil in 1993 (holes 3 and 10; **Fig 1B**) and again in 2006 (holes 13, 20, and 21; **Fig 1C**), indicating long-term presence through time (persistence) of this lineage for over a decade. In addition, ST617 also was recovered from a goat sampled at a different farm 25 km away from the study site. This is interesting as ST617 has been an uncommon ST in the Northern Territory and has not been found elsewhere in Australia or globally, according to the pubMLST database [87]. However, this obviously does not preclude it from being common but unsampled within this local area. Soil isolates from ST617 were obtained primarily near the shed complex in the western enclosure in both soil studies, but also from a single hole (21) at the northwestern corner of the male enclosure in 2006 (**Fig 1**).

The different *B*. *pseudomallei* STs recovered from soil exhibited dissimilar spatial distributions and temporal patterns across the study site (**Fig 1** and **S1 Table**). ST260 showed a very limited distribution and was only found in soil study #1 (1993–1994). ST260 isolates were obtained from the northeastern corner of the shed complex (**Fig 1B**) in soil samples from hole 1 (40 cm) and hole 2 (20, 60, and 90 cm). We recovered only one ST260 isolate (MSHR0225) from the goats that were sampled throughout the 1990s. ST266 was also uncommon but detected in both soil studies. ST266 isolates were obtained from shallow soil samples (10–20 cm) at hole 1 in both 1993 and 1994 (**Fig 1B**), and in 2006 from hole 44 (10 cm) in the dry area to the south of the goat enclosures (**Fig 1C**). ST266 was associated with severe illness in two goats (goat isolates MSHR0403 and MSHR0864; **S1 Table**) and causes melioidosis in humans [88]. All ST326 isolates were collected in 2006 from the northern area of the sampling grid from holes located in both goat enclosures and the irrigated walkway between them (holes 2, 14, 20, 22, 24, 33 and 35; **Fig 1C**). Although ST326 was not detected in soil study #1, by 2006 it had become the most common ST in soil (47 of 62 genomes from soil study #2; **S1 Table**). In contrast, ST617 showed a limited distribution in soil and was found only in the northern holes of goat enclosures; however, it was the most common ST in goats (17 of 23 genomes; **S1 Table**). Soils positive for ST617 were obtained from the shed complex at 20 cm depths in holes 3 and 10 in 1993 (**Fig 1B**), and again in 2006 at 30 cm in holes 13, 20, and 21 (**Fig 1C**). Other STs from soil were only identified from single samples during soil study #1 (ST109, ST320, ST714; **Fig 1B**) or soil study #2 (ST1488; **Fig 1C**). ST109 is widespread in Australia and frequently infects human patients [88] (**Table 2**) but, surprisingly, it did not cause goat infections at this farm. This is perhaps because this ST was rare at the study site, being found in only two soil samples (20 and 40 cm) collected from hole 5 on one sampling date, 24 March 1993 (**S1 Table**).

**Fig 3:**
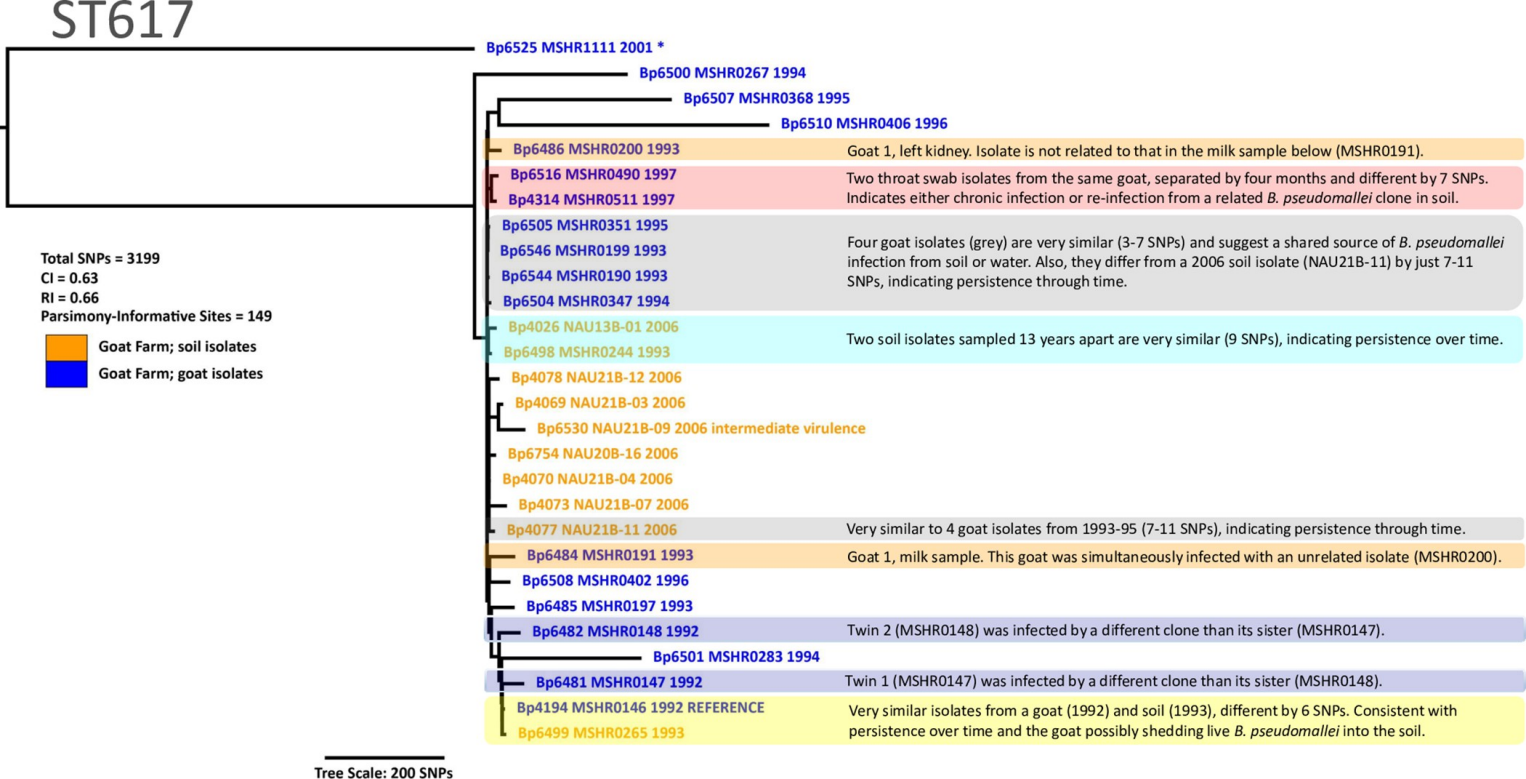
Maximum Parsimony phylogeny of *B*. *pseudomallei* isolates from this study assigned to the phylogenetic group corresponding to ST617. To maximize the proportion of each genome used to call SNPs, raw reads were aligned against an internal high-quality genome reference (Bp4195 MSHR0146 1992 REFERENCE) that resulted in 6,751,687 shared positions among these 28 genomes, from which 3,199 total SNPs were identified (149 were parsimony-informative). An asterisk (*) indicates the genome used to root the tree; blue font indicates isolates obtained from goats; orange font indicates isolates obtained from soil.

Focused genomic analyses within each of the four most common STs present at the goat farm (ST260, ST266, ST326, ST617) provided additional insights into the specific phylogenetic relationships among soil and goat isolates assigned to these STs (**Figs 3** and **S2**). Because these analyses utilized a high-quality reference genome from within each ST group, there was a high proportion of orthologous regions shared by the reference and the other genomes within each of these STs (*i*.*e*., corresponding to 86–96% of the reference genomes used in these intra-ST comparisons compared to 48% of the reference genome in the analysis used to construct the global phylogeny in **Fig 2**). This resulted in the identification of additional SNPs that would not have been detected when using a less related reference genome, thereby facilitating a fine-scale examination of phylogenetic relationships among highly similar isolates.

Within ST617 we found evidence for several possible epidemiological links based on phylogenetic comparisons (**Fig 3**), some of which had been reported previously using 21 variable number tandem repeat (VNTR) markers [49]. Isolates MSHR0490 and MSHR0511 were cultured from two throat swabs collected four months apart from a single goat (14 March1997 and 29 July 1997); the two genomes differ by seven SNPs (**S5 Table**). This close similarity most likely resulted from a persistent infection in the goat, but an alternative explanation is that the goat was re-infected by a very similar *B*. *pseudomallei* clone present in contaminated soil or water. Likewise, we found four other ST617 isolates (MSHR0190, MSHR0199, MSHR0347, MSHR0351) that were closely related, differing by just 3–7 SNPs (**Fig 3**). This group of isolates is particularly interesting because MSHR0199 is a goat isolate obtained from a different farm located 25 km distant. The other three isolates were obtained from goats at the study farm from 1993 to 1995 and provide evidence that *B*. *pseudomallei* was present for two years and caused multiple infections in goats with little genomic change. All three of these isolates were sampled from the western enclosure and demonstrate the possibility of amplification within a host followed by persistence through time in the environment. Indeed, these four goat isolates were found to be similar to a 2006 soil isolate (NAU21B-11) that is different from these goat isolates by just 7–11 SNPs, consistent with long-term presence in the environment. In a third example from ST617, we identified a soil isolate from 1993 (MSHR0265; hole #10, 20cm) that differed from a goat isolate obtained in 1992 (MSHR0146) by just six SNPs (**S5 Table**). Because the goat isolate was collected first, the close genomic similarity of these two isolates suggests the infected goat may possibly have shed live *B*. *pseudomallei* into the soil, which was then recovered the following year from hole #10 in the shed complex in the western enclosure (**Fig 1B**). A number of other soil isolates collected in 2006 suggest environmental persistence given their high genetic similarity with isolates collected in the 1990s (**S5 Table**). One example is NAU13B-01 (2006 soil), which differs from MSHR0244 (1993 soil) by 9 SNPs. This type of focused SNP-based analysis provides a powerful way to infer genetic connections because each pairwise comparison utilized a closely-related ST617 reference genome (MSHR0146), hence a higher percentage of actual SNPs in each genome were identified.

Whole genome comparisons within ST617 also provided insights about isolates that were less closely related. A single female goat infected with *B*. *pseudomallei* yielded two isolates collected on the same day (01 April 1993); one was obtained from milk (MSHR0191) and the other from the goat’s kidney (MSHR0200), and the isolates differed by 21 SNPs (**Fig 3**). This suggests either a long-term infection of this goat that allowed time for independent mutations to accumulate, or that the goat was simultaneously co-infected by different *B*. *pseudomallei* clones. We also sampled two different pairs of twin goats that contracted melioidosis. In the first pair, twin females were both positive on 10 November 1992 and the *B*. *pseudomallei* isolates obtained from them (MSHR0147 and MSHR0148) differed by 23 SNPs. The other twin females were positive on 10 November 1992 and 04 February 1994, respectively, and the isolates obtained from them (MSHR0146 and MSHR0283) differed by 17 SNPs. The occurrence of distinct *B*. *pseudomallei* genomes in individual goats is not surprising, because each twin was likely infected independently from the environment by distinct *B*. *pseudomallei* lineages. Finally, we note three isolates in ST617 that stand out as being distinct with no close genetic connections to any other isolates included in these analyses: MSHR0368 (35–77 SNPs between it and any other isolate), MSHR0406 (46–77 SNPs), and MSHR0276 (253–285 SNPs). These distinct isolates illustrate the significant diversity that is present within this phylogenetic group and this site.

The ability to compare genomes within ST260, ST266, and ST326 revealed additional insights within each ST group. The one ST260 isolate obtained from an infected goat in 1993 (MSHR0225) differed from the four soil isolates also collected in 1993 by 93–117 SNPs and was clearly from a distinct phylogenetic lineage (**S2A Fig**). From this we may infer the presence of additional diversity within ST260 that was not sampled during either soil study. Likewise, all analyzed ST266 isolates were quite distinct from each other (**S2B Fig**). The two goat isolates assigned to ST266 (MSHR0403 and MSHR0864) were not closely related and differed by 717 SNPs (**S5 Table**). Of the two, MSHR0864 had closer similarity to isolates from soil (59–116 SNPs), but we did not identify any obvious genetic connections. Comparisons among ST266 soil isolates did not reveal any highly similar genomes; we found 72 SNPs between two soil isolates from 1993–1994 (MSHR0226 and MSHR0294) and 42–143 SNPs among the five 2006 soil isolates obtained from hole 44 (**S5 Table**).

In stark contrast to patterns within ST260 and ST266, most ST326 isolates were closely related (**S2C Fig**), with 43 of 47 genomes differing by only 0–28 SNPs (**S5 Table**). Two soil samples, NAU14A and NAU35A, both yielded a small number of highly similar ST326 genomes differing by just one SNP (**S5 Table**), indicating that we likely had isolated clones present within these soil samples. We also found evidence of a clonal lineage that was isolated from multiple holes located within a ~30-meter^2^ area. This ST326 clone was represented by a set of five isolates with 0–1 SNPs in their pairwise genome comparisons (**S5 Table**). Two of these isolates (NAU14A-08 and NAU14B-10) were obtained from hole 14 and had no SNP differences between them. The other three (NAU20B-10, NAU24B-03, and NAU35A-01), which were isolated from holes 20, 24, and 35 (Fig 1C), also had no SNP differences among them and were different from the two hole 14 isolates by only a single SNP. Taken together, these five nearly identical genomes were obtained from four different holes located in both goat enclosures and the irrigated walkway (Fig 1C), possibly indicating a rapid spread of ST326 leading up to 2006 (**S2C Fig**). At the other extreme, three ST326 genomes were highly diverse compared to all others. NAU2B-10 differed from the other ST326 isolates by hundreds of SNPs and clearly originated from a distinct ST326 lineage. In addition, isolates NAU33A-03 and NAU33A-07 both yielded very long branch lengths in the phylogeny, which is an indication they may have recombined with unrelated *B*. *pseudomallei* genomes from other STs [36]. No other ST326 isolates were similar to either of these two isolates, and these unique recombinants may not have been widespread at the time of the 2006 soil study.

### *In vivo* animal challenge

**Fig 4:**
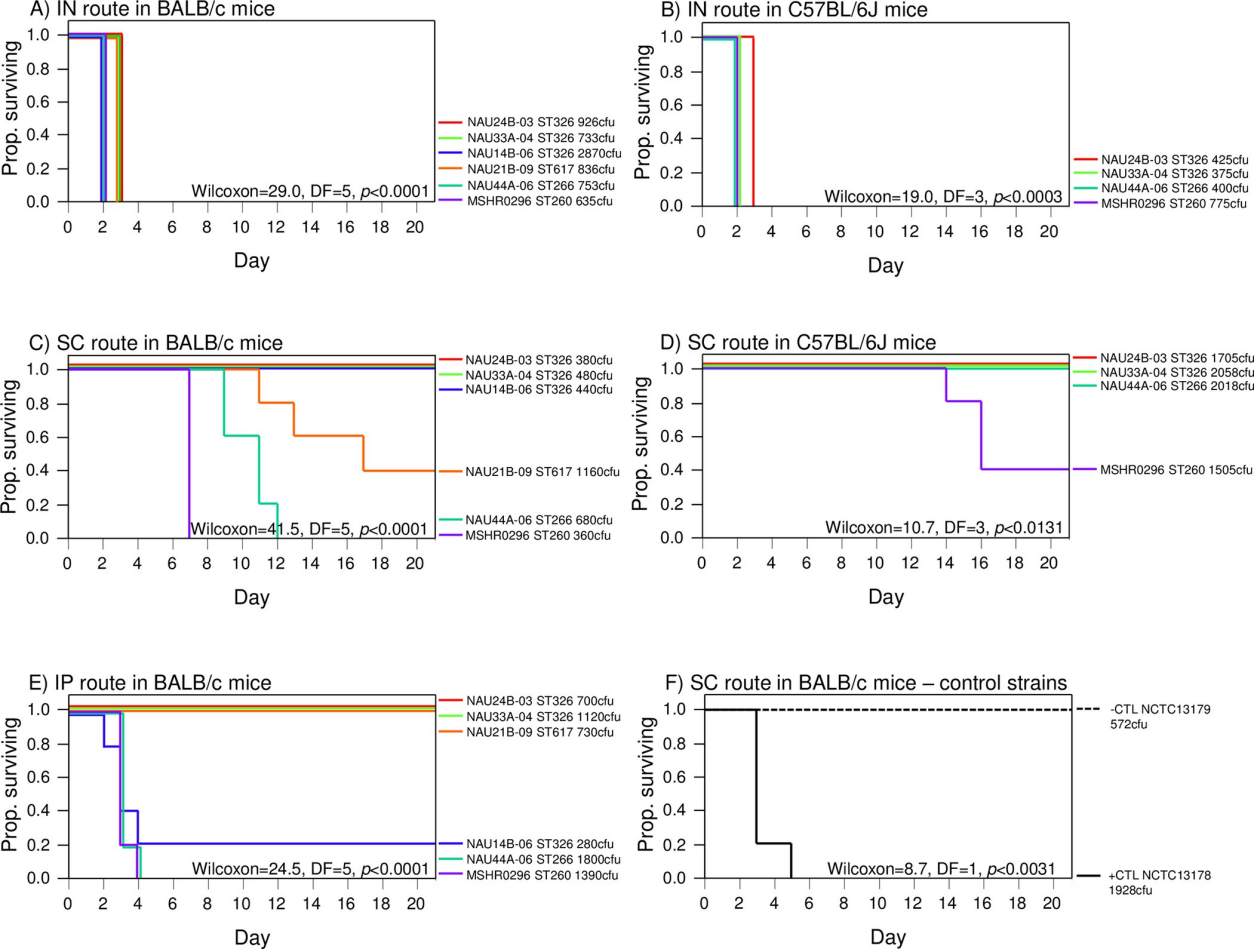
Survival plots for BALB/c and C57BL/6J mice challenged with *B*. *pseudomallei* isolates collected from soil at our study site in Australia. **Routes of infection were IN (intranasal), SC (subcutaneous), and IP (intraperitoneal).** The color assigned to the line for each isolate is the same in all plots. The injected dose for each isolate is provided after each isolate name and was estimated by counting colony forming units (cfu) on dilution plates for each inoculum. **A)** IN route in BALB/c mice using six isolates; **B)** IN route in C57BL/6J mice using four isolates; **C)** SC route in BALB/c mice using six isolates; **D)** SC route in C57BL/6J mice using four isolates; **E)** IP route in BALB/c mice using six isolates; **F)** SC route with fully virulent (NCTC13178) and attenuated (NCTC13179) control isolates in BALB/c mice.

The BALB/c and C57BL/6J mouse challenge studies identified two soil isolates, MSHR0296 (ST260) and NAU44A-06 (ST266), that were highly virulent and killed all mice in every experiment but one (**Fig 4**). The IN route stood out as being the most lethal (**Fig 4A** and **4B**), with all isolates killing all mice by day 2–3. In addition, the five mice in each group died earlier after IN challenge compared to the other routes (Wilcoxson = 68.5, DF = 2, P<0.0001). In contrast, the SC route (**Fig 4C** and **4D**) was not as lethal as IN and we observed greater variation in virulence across *B*. *pseudomallei* isolates and mouse strains using this route. MSHR0296 and NAU44A-06 killed all BALB/c mice via the SC route, although the time to death was longer than for IN. NAU21B-09 (ST617) showed an intermediate level of virulence via the SC route and killed three of five BALB/c mice. C57BL/6J mice were much less susceptible to the SC route and only three mice died in total, all of which were infected with MSHR0296. Using the IP route, MSHR0296 and NAU44A-06 were again highly virulent and killed all BALB/c mice by day four (**Fig 4E**). A third soil isolate, NAU14B-06 (ST326), also showed high virulence and killed four of five BALB/c mice by day four. Two of the soil isolates, NAU24B-03 and NAU33A-04 (both ST326), were much less virulent and did not kill any BALB/c mice in the SC or IP trials. The virulent and attenuated controls (NCTC13178 and NCTC13179, respectively) performed as expected in BALB/c mice (**Fig 4F**). All survival data and clinical scores are provided in **S6 Table**.

### Conservation of genes associated with virulence

Our screen of 552 potential virulence factors in *B*. *pseudomallei* (S3 Table in Sahl et al. [86]) across the 92 genomes generated in this study identified three loci that were always present in genomes from the two high virulence STs (ST260 or ST266) but absent from 45 of 47 genomes from the low virulence ST326 (**S7 Table**). In our study, these STs represent the extremes for high vs. low virulence. Lactonase family protein A (*lpfA*; BPSS2074) was present in all genomes from ST260, ST266, and ST617 but only two of 47 genomes from ST326 (NAU2B-10 and NAU22A-05). The oxoacyl ACP synthase locus (BMA2880) was present in all genomes from ST260, ST266, and two of 27 ST617 genomes (MSHR0283 and NAU21B-09), but absent from all ST326 genomes. The adhesion locus *fha*B1 was present in all genomes from ST260 and ST266. However, this gene appears to have been slightly truncated in eight of nine ST266 genomes because the breadth of coverage was <90% (**S7 Table**); this included the ST266 strain that showed high virulence in mice (NAU44A-06). The *fha*B1 locus was also partially truncated in all ST617 genomes and two ST326 genomes (NAU2B-10 and NAU22A-05) and absent in the remaining ST326 genomes. A fourth locus, aldehyde dehydrogenase (BMAA0630) exhibited the opposite pattern: it was extremely truncated in all genomes of ST260 and ST266 except one 1999 goat isolate (MSHR0864), and fully present in all ST617 and ST326genomes. Finally, we analyzed the 12 loci that encode LPSA (loci BPSL2675 *wbi*F through BPSL2684 *rml*C dTDP), which were present in all genomes of ST266, ST617, and ST326, but not in ST260, which carries the LPSB variant. None of the other virulence loci revealed strong patterns of presence/absence across STs. For example, the Type IV secretory system locus *hcp1* (BPSS1498), which has previously been linked with virulence in *B*. *pseudomallei* [89], was present in all 92 genomes.

## Discussion

This study provides a demonstration of the striking genomic variation that *B*. *pseudomallei* can display on even a small spatial scale. By sequencing the genomes of a large number of isolates collected from goats and soil, we provide a multi-faceted investigation of goat melioidosis at this farm. This is one of the highest concentrations of livestock melioidosis cases known for Australia, aside from intensive piggery operations in Queensland [21,90]. Multiple phylogenetic lineages of *B*. *pseudomallei* present in soil were associated with severe disease in goats. Twelve Australian STs were present at the study site and of the 11 STs that were previously reported, nine have been documented to cause melioidosis in humans. This diversity is similar to that found in other studies that have documented high genomic diversity in *B*. *pseudomallei* at multiple spatial scales [91,92], from a global perspective [38] down to within a single soil sample collected in Thailand [48]. Fortunately, no human cases of melioidosis occurred at this site, despite the prevalence of *B*. *pseudomallei* in goats and soil in the shed complex where goats gathered for food, water, and shade. This likely reflects that the owners were healthy and therefore at decreased risk for melioidosis compared to those with clinical risk factors [9]. In addition, they wore robust footwear and practiced good animal husbandry, including hand hygiene when milking the goats. Such practices emphasize the importance of public health policy and education efforts for reducing the risk of melioidosis in humans.

Given the number of female and kid goats that contracted melioidosis, it is not surprising that *B*. *pseudomallei* was frequently encountered in soil within the western enclosure. We hypothesize that conditions at this agricultural operation may have led to an accelerated transmission cycle between soil and goats. The presence of a goat herd and other factors such as irrigation and protection from UV radiation under shade structures and trees appear to have been favorable for certain STs, including ST617, ST260, and ST266. At different times, each of these STs began to independently infect goats. Once infected, the goats would have amplified *B*. *pseudomallei* and could possibly have inoculated new locations in soil via live cells excreted in urine, essentially seeding a wider area with viable *B*. *pseudomallei* that could then infect new goats in a local animal-environment transmission cycle. Live *B*. *pseudomallei* cells are readily shed in urine from humans and animals [93–95], which feasibly could persist at the soil surface long enough to infect other hosts. The genomic patterns we observed support the possibility of local cycling between goats and soil; however, this hypothesis remains untested. The ST617 lineage appears to have been particularly successful at infecting goats and represented 74% of the goat isolates that we analyzed. Closely related ST617 genomes were found in a number of goat samples, providing evidence that at least some goats were likely infected from a single clone of *B*. *pseudomallei* (or at least closely related clones) at different timepoints. The combination of amplification in hosts and persistence in soil was clearly associated with an elevated risk of exposure to *B*. *pseudomallei* at this location. Melioidosis became such an important problem that all goats were removed from the farm by 2004, 12 years after the first described cases in 1992.

Our study contributes to a better understanding of the diverse STs that can cause melioidosis. Three STs (ST260, ST266, and ST617) were found in both goats and soil, and four others (ST131, ST328, ST1356, and ST1986) were each detected only once in goats (Table 2). Although they may have also been present in soil, our sampling techniques did not allow us to detect these STs in either soil study. This matches a pattern for human cases wherein the detection of many “singleton” STs has been observed [40,96]. Despite being rarely encountered elsewhere in northern Australia, isolates assigned to ST617 caused the vast majority of goat infections at this farm. This may indicate that ST617 isolates at this site were more common in soil and/or more successful at infecting goats. Alternatively, it is also possible that random chance led to the majority of goats being infected by ST617, and that other STs could easily have done the same. However, the ST617 isolate MSHR0511 has been shown to cause acute disease in goats experimentally challenged with medium doses via aerosol (1.1–2.3 x 10^4^ cfu) and percutaneous (7.7–9.9 x 10^3^ cfu) routes of infection [51,52]. In this study we also report a ST617 isolate (MSHR0199) obtained from an infected goat at a farm 25 km away from the study site, which suggests this lineage is distributed regionally but perhaps has not been dispersed widely in northern Australia. ST617 is very rarely reported in the environment elsewhere in Australia [50] and has only been associated with one human case (https://pubmlst.org/organisms/burkholderia-pseudomallei). This patient was a 30-year-old woman with risk factors for melioidosis who presented with melioidosis pneumonia and septic shock requiring intensive care management, including ventilation, but she survived. In northern Australia, most STs show a similar pattern of limited geographic dispersal [97]. Several other STs known to cause melioidosis in humans were recovered from soil samples at our study site, including ST109, ST266, ST320, and ST714 (Table 2). The absence of goat infections caused by ST109 was rather surprising, given that it is the most prevalent ST in human infections and widespread in the Darwin area [31].

It is possible that certain lineages of *B*. *pseudomallei* might differentially benefit from a close association with goats and modified soil. Two STs isolated from goats (ST617 and ST266) persisted at this site for over 10 years. ST617 was encountered in multiple soil samples next to (and inside) the shed complex of the western enclosure. Whole genome sequencing data revealed surprisingly little genetic differentiation over time in ST617, with only 8–9 SNPs separating some of the soil isolates sampled 13 years apart (S5 Table). ST266 isolates were also present in soil at the shed complex in 1993–1994, but in 2006 we found this ST only in the drier (non-irrigated) area to the south, downslope of the goat enclosures. A greater level of differentiation was observed in ST266, with at least 64 SNPs separating isolates sampled 12–13 years apart (S5 Table). Detecting ST266 and ST617 through time is important because it demonstrates the long-term presence of infectious lineages of *B*. *pseudomallei* within soil, even in the absence of mammalian hosts, which demonstrates the potential risk in agricultural operations at other locations.

By 2006, ST326 was by far the most widespread and abundant ST at the study site. This was somewhat surprising because this ST had not been identified during the first soil study in 1993–1994. It is possible that ST326 was present during soil study #1 but simply missed due to our sampling being focused on and around the shed complex in that first study, where lineages that infected goats might be expected to dominate. Furthermore, our ability to detect rare ST326 isolates was reduced because we generated ST data for only 11 of the 33 isolates from soil study #1 (S1 Table). Alternatively, it is possible that ST326 was absent from this site while goats were present but colonized the area later and spread by 2006. This lineage was not detected in goats and had lower virulence in our mouse challenge study, which suggests that any ST326 lineages present were not highly infectious. ST326 is known from other environmental samples in northern Australia [50] but has only been reported in one human patient–a mild cutaneous infection following environmental exposure of a lacerated foot in a healthy 7 year old boy without systemic illness; he lived in a rural Darwin location 25 km from the goat farm [96]. Genome sequences of ST326 isolates at our study site support a scenario in which a small number of founding genotypes may have been present, but one clone was clearly more successful than the others (S2 Fig). If ST326 was present but rare, it apparently did not amplify and spread in the way of other lineages that infected goats (ST617, ST260, and ST266). Competition with these other lineages was possibly higher when goats were present, which would suggest that host presence has a differential influence on various phylogenetic lineages of *B*. *pseudomallei*.

In the 2006 soil study, *B*. *pseudomallei* was found primarily in holes 1–37 from the northern area of the study site that encompassed the goat enclosures and the grassy walkway between them. This area was regularly irrigated and contained more ground vegetation than the drier, open area to the south. Irrigation has previously been shown to be an important determinant for *B*. *pseudomallei* occurrence in gardens [33,98]. Among the specific soil measurements we evaluated, the ratio of total nitrogen to organic matter was most strongly associated with *B*. *pseudomallei* presence across the entire sampling grid. This is also consistent with a negative association between *B*. *pseudomallei* and carbon to nitrogen ratio that has previously been shown in other studies [57,99,100]. Considering the greater concentration of *B*. *pseudomallei* positive holes in the northern area, we performed an *a posteriori* analysis of abiotic soil variables in the northern versus southern areas and found the soil in the northern area had significantly higher moisture, contained more nitrogen and organic matter, and was more saline. Although these findings are valuable, we acknowledge that a more precise understanding of *B*. *pseudomallei* occurrence and persistence in the environment requires further investigation.

Regardless of location, *B*. *pseudomallei* was more likely to occur in soil at a depth of 30 cm compared to 10 cm. Most 30 cm samples were reddish or orange-brown soil, which has been associated with *B*. *pseudomallei* presence [33]. The 30 cm samples also contained less moisture on average than 10 cm samples, probably due to the presence of grass and other vegetation that might have prevented irrigation water from draining down to 30 cm during the late dry season, when soil study #2 was conducted. At first glance, this seems counter-intuitive for explaining why we found more *B*. *pseudomallei* at 30 cm. However, in the long term this deeper soil would provide a more stable environment with less variation in water content and has a lower risk of extreme desiccation than soil closer to the surface. In addition, some bacteriophages specific to *B*. *pseudomallei* are activated as temperatures increase at the surface, which could possibly become a selective pressure that reduces the population of viable *B*. *pseudomallei* cells at the top layer of soil [101]. The ability of *B*. *pseudomallei* to persist at 30 cm and deeper soil layers is well documented [33,102], and consistent with the hypothesis that suggests *B*. *pseudomallei* persists in deeper soils at the upper edge of the water table, and then migrates upwards opportunistically during the wet season [102,103].

In the *in vivo* challenge study, the IN route stood out as being remarkably lethal in all BALB/c and C57BL/6J mice, regardless of *B*. *pseudomallei* isolate or ST. This is not surprising, given the increased severity of respiratory melioidosis acquired via inhalation or aspiration [83]. Another significant finding was that two soil isolates, MSHR296 (ST260) and NAU44A-06 (ST266), were highly virulent across all three infection routes; these isolates originated from distinct phylogenetic lineages that also had been cultured from internal organs of moribund goats (S1 Table). The remaining three isolates from ST326 and one from ST617 revealed differential virulence in the IP and SC infection models, documenting that closely-related virulent and non-virulent *B*. *pseudomallei* can occur at a single location, consistent with previous findings [48]. The mouse challenge study provides important experimental results supporting the hypothesis that, at least in a mouse model, some isolates of *B*. *pseudomallei* can cause severe disease whereas others result in milder symptoms.

The variability in virulence among *B*. *pseudomallei* isolates obtained in this study provided an opportunity to compare those phenotypic variations with putative virulence loci that were variably present in these isolates. We identified one locus, oxoacyl ACP synthase (BMA2880), that was present in all genomes from ST260 and ST266, variably present in ST617, and absent from all ST326 genomes. In contrast, *hcp1*, a locus associated with virulence in *B*. *pseudomallei* isolates from Thailand [48], was present in all high and low virulence isolates in our study. We note that ST326 is known to have caused one human case of percutaneous melioidosis in the Darwin region [96], which might suggest that the infecting ST326 isolate had acquired a needed set of virulence loci through recombination. Acquisition of virulence loci and other genomic components via horizontal gene transfer is hypothesized to be a common occurrence in *B*. *pseudomallei* [6,37,47]. Taken together, our study identifies variably present loci that may allow certain genotypes to cause melioidosis in humans and other animals. However, it is possible that certain gene combinations are only important at this site or in this specific combination of soil and goat hosts.

## Conclusions

This study provides insight into the importance of more fully understanding habitats that increase the abundance and presence of *B*. *pseudomallei* in soils, which in turn leads to an increased risk of melioidosis in humans and agricultural animals. Our study identified specific soil factors that appear to be associated with increased *B*. *pseudomallei* occurrence and, thus, severely impact the production of agricultural animals. Although diverse lineages of *B*. *pseudomallei* may exist in a given environment, some appear to colonize mammals more efficiently. The broad genomic diversity and ability to infect many host species are important characteristics of this One Health pathogen. These results have One Health implications for environmental surveys on risk assessment as well as understanding the drivers of pathogenesis in environmental *B*. *pseudomallei* strains. There remains a need for long-term soil monitoring that incorporates multiple data types (*e*.*g*., climate, vegetation, composition of soil microbiome, soil chemical profiles, and *B*. *pseudomallei* genomics) to fully understand the risk of acquiring melioidosis from the environment [104]. *Burkholderia pseudomallei* is an emerging pathogen in traditionally non-endemic areas, and management of this disease will benefit from monitoring in the context of a One Health surveillance framework.

## Supporting information

S1 Fig In soil study #2 (2006), there was a positive spatial autocorrelation of *Burkholderia pseudomallei* occurrence at the Australian study site (Fig 1C) for holes closer than 30 meters (Moran’s I, permutation test p<0.05). **A)** Semivariogram plot showing the level of variation in *B*. *pseudomallei* occurrence among holes within each distance interval (meters). **B)** plot of Moran’s I. “NS” not significant. The spatial autocorrelation is likely due to irrigation and the presence of goats in the northern area of the sampling grid, where *B*. *pseudomallei* was detected in 21 of 36 holes (58%).
(TIF)

S2 Fig Individual maximum parsimony phylogenies of *Burkholderia pseudomallei* isolated from ST groups ST260, ST266, and ST326. Blue font indicates goat isolates, orange font indicates soil isolates. To maximize the percent coverage of each genome used to call SNPs, raw reads were aligned against a high-quality genome reference chosen within each ST group (labelled as “REFERENCE” in each tree). An asterisk (*) indicates the genome used to root each individual tree. Genome coverage was high in all individual trees: **ST260)** 862 total SNPs based on 93% coverage of reference genome MSHR0227; **ST266**) 1,503 total SNPs based on 96% coverage of reference genome NAU44A-06; **ST326)** 2,361 total SNPs based on 86% coverage of reference genome NAU35A-03.
(TIF)

S1 Table Complete list of all *Burkholderia pseudomallei* isolates (n = 195) cultured from goats, soil study #1, and soil study #2. We chose 92 *B*. *pseudomallei* isolates for whole genome sequencing (listed as "Y" under WGS column), six of which were used in a mouse challenge study (noted under Column H). Nine of the isolates not chosen for WGS had been independently sequenced at seven MLST loci and uploaded to pubMLST for *B*. *pseudomallei* (MSHR0124, MSHR0212, MSHR0215, MSHR0219, MSHR0231, MSHR0294, MSHR0298, MSHR0408, and MSHR0484).
(XLSX)

S2 Table Complete list of all samples from soil study #1 (128 samples) and soil study #2 (100 samples) collected at the goat farm study site (Fig 1). The most complete data for soil variables were generated during soil study #2. Presence of *Burkholderia pseudomallei* (*Bps*) was determined either by culturing from soil (Column D) or real-time PCR using DNA extractions from 500 mg of soil (Column E). The legend for each soil variable is provided in a separate worksheet tab (Legend for Soil Variables).
(XLSX)

S3 Table GenBank accession numbers for whole genome sequences of 92 *Burkholderia pseudomallei* isolates from this study, plus 693 publicly available genomes in GenBank used for the global phylogeny (Fig 2). A subset of assembly accessions (Column F) were not submitted because these genomes showed high similarity to others with the same MLST.
(XLSX)

S4 Table Comparison of abiotic variables from soil study #2 within the context of two important site factors (grid area and sample depth) that were associated with *B*. *pseudomallei* occurrence at our study site (Table 1). Within both site factors, numerical variables are listed first and reported as median and min-max range. Binary variables (yes/no) are listed second, and reported as the percentage of samples that were in the “yes” category (n) compared to the total samples possible (n_tot_) within that category (n/n_tot_). Each numerical and binary predictor was analyzed with the odds ratio (OR) statistic; only P-values are reported. **A)** Comparison of samples from two grid areas (northern versus southern). The northern area includes holes 1–36 sampled from irrigated goat enclosures and the grassy walkway between them (see **Fig 2**); this area was the source of nearly all *B*. *pseudomallei*-positive soil samples in 2006. **B)** Comparison of soils sampled at depths of 10 cm versus 30 cm throughout the study site.
(DOCX)

S5 Table Pairwise SNP matrix for genomes of *Burkholderia pseudomallei* isolates from goats (blue font) and soil (orange font). SNPs were identified via comparison to a high quality reference genome within each multi-locus sequence type (MLST) group.
(XLSX)

S6 Table Survival and clinical data from the mouse challenge study using six isolates of *Burkholderia pseudomallei*. Infection routes for BALB/c mice included subcutaneous (SC), intraperitoneal (IP), and intranasal (IN). As a comparison of mouse models, we also challenged C57BL/6J mice using four of the six isolates in the SC and IN routes. Each combination of mouse/isolate/infection route (five total) is shown on a separate worksheet.
(XLSX)

S7 Table List of 21 virulence loci of 552 loci screened across 92 *Burkholderia pseudomallei* genomes from this study (S3 Table). The sequence reads for each locus were tested for breadth of coverage against reference genome K96243 and the percentage recorded.
(XLSX)
