# Supplementary figures and images for: Melioidosis in goats at a single Australian farm was caused by multiple diverse lineages of Burkholderia pseudomallei present in soil
Source: PLoS Negl Trop Dis. 2024 Dec 19;18(12):e0012683. doi: 10.1371/journal.pntd.0012683 (PMC11698569; doi:10.1371/journal.pntd.0012683)

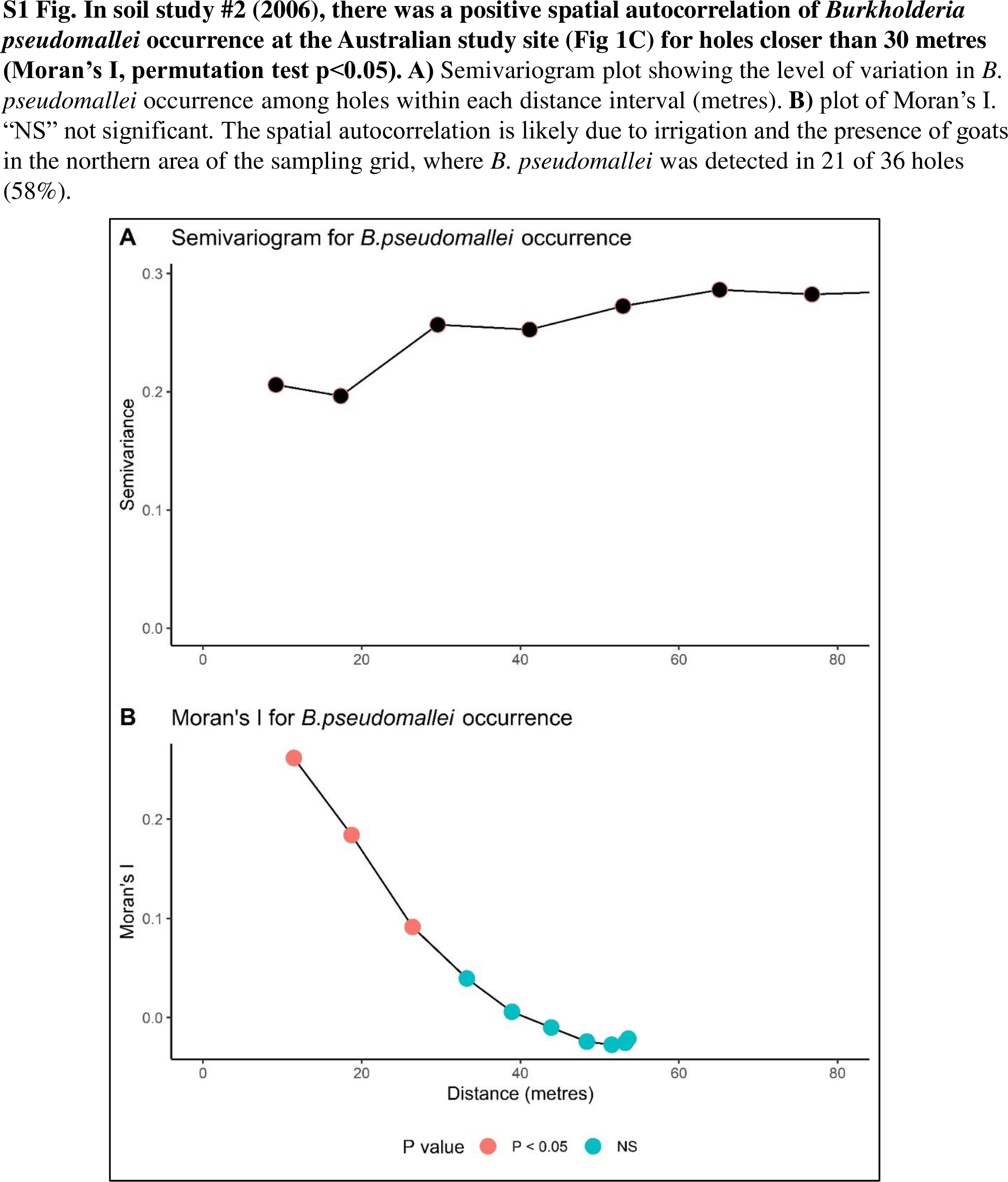

Supplement: S1 Fig — A) Semivariogram plot showing the level of variation in B. pseudomallei occurrence among holes within each distance interval (meters). B) plot of Moran’s I. “NS” not significant. The spatial autocorrelation is likely due to irrigation and the presence of goats in the northern area of the sampling grid, where B. pseudomallei was detected in 21 of 36 holes (58%). (TIF) [file pntd.0012683.s001.tif]

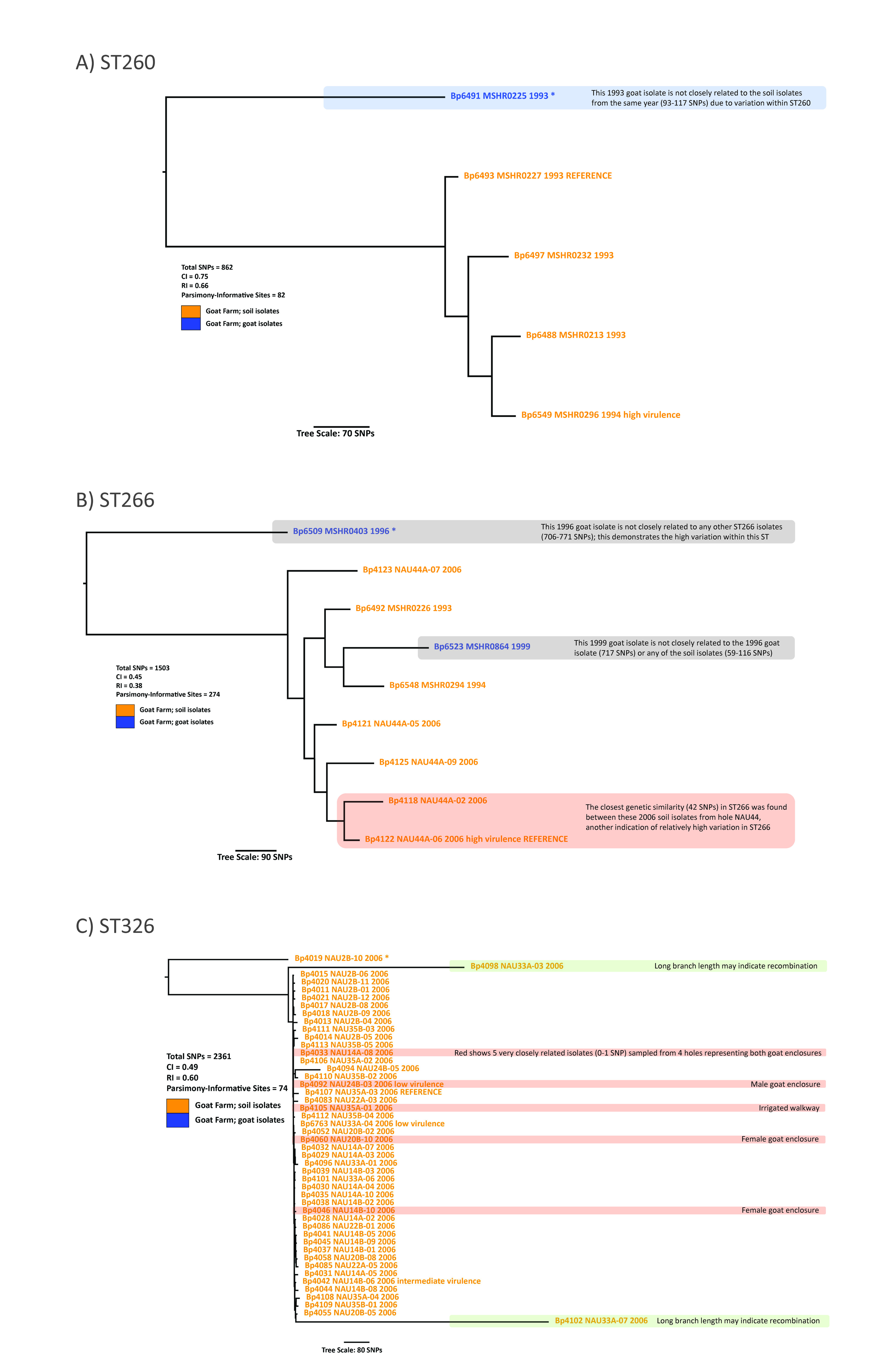

Supplement: S2 Fig — Blue font indicates goat isolates, orange font indicates soil isolates. To maximize the percent coverage of each genome used to call SNPs, raw reads were aligned against a high-quality genome reference chosen within each ST group (labelled as “REFERENCE” in each tree). An asterisk (*) indicates the genome used to root each individual tree. Genome coverage was high in all individual trees: ST260) 862 total SNPs based on 93% coverage of reference genome MSHR0227; ST266) 1,503 total SNPs based on 96% coverage of reference genome NAU44A-06; ST326) 2,361 total SNPs based on 86% coverage of reference genome NAU35A-03. (TIF) [file pntd.0012683.s002.tif]
