# Supplementary material for: Melioidosis in goats at a single Australian farm was caused by multiple diverse lineages of Burkholderia pseudomallei present in soil
Source: PLoS Negl Trop Dis. 2024 Dec 19;18(12):e0012683. doi: 10.1371/journal.pntd.0012683 (PMC11698569; doi:10.1371/journal.pntd.0012683)
Supplement: S4 Table — Within both site factors, numerical variables are listed first and reported as median and min-max range. Binary variables (yes/no) are listed second, and reported as the percentage of samples that were in the “yes” category (n) compared to the total samples possible (ntot) within that category (n/ntot). Each numerical and binary predictor was analyzed with the odds ratio (OR) statistic; only P-values are reported. A) Comparison of samples from two grid areas (northern versus southern). The northern area includes holes 1–36 sampled from irrigated goat enclosures and the grassy walkway between them (see Fig 2); this area was the source of nearly all B. pseudomallei-positive soil samples in 2006. B) Comparison of soils sampled at depths of 10 cm versus 30 cm throughout the study site. (DOCX) [file pntd.0012683.s006.docx]

**S4 Table. Comparison of abiotic variables from soil study #2 within the context of two important site factors (grid area and sample depth) that were associated with *B. pseudomallei* occurrence at our study site (Table 1).** Within both site factors, numerical variables are listed first and reported as median and min-max range. Binary variables (yes/no) are listed second, and reported as the percentage of samples that were in the “yes” category (n) compared to the total samples possible (n_tot_) within that category (n/n_tot_). Each numerical and binary predictor was analyzed with the odds ratio (OR) statistic; only P-values are reported. **A)** Comparison of samples from two grid areas (northern versus southern). The northern area includes holes 1-36 sampled from irrigated goat enclosures and the grassy walkway between them (see **Fig 2**); this area was the source of nearly all *B. pseudomallei*-positive soil samples in 2006. **B)** Comparison of soils sampled at depths of 10 cm versus 30 cm throughout the study site.

| **Soil variables** | **Median (min-max) for numerical**  **and % yes (n/n_tot_) for binary** | | **^a^P-value** |
| --- | --- | --- | --- |
| **A) Northern vs. southern area** | **Northern** | **Southern** |  |
| Soil pH | 5.8 (5.4-6.7) | 5.4 (4.8-5.8) | <0.001 |
| Moisture (% vsw) | 16.5 (9.0-24.0) | 13.0 (7.0-20.0) | <0.01 |
| **^b^**Conductivity (salinity) (uS/cm) | 53.0 (11.0-419.0) | 21.5 (9.0-64.0) | <0.001 |
| Total N (%) | 0.14 (0-0.62) | 0.05 (0-0.17) | <0.001 |
| Organic C (%) | 6.29 (2.2-15.9) | 4.02 (2.2-7.1) | <0.001 |
| Ratio total N/organic C (x 10e3) | 21.2 (0-63.4) | 14.4 (0-24.0) | <0.001 |
| Gravel (%) | 0 (0-27.5) | 0 (0-27.5) | 0.45 |
| Red/orange soil color (Y, N) | 27.8% (20/72) | 50.0% (14/28) | <0.001 |
| Clay (Y, N) | 54.2% (39/72) | 57.1% (16/28) | 0.80 |
| Vegetation (Y, N) | 91.7% (66/72) | 0% (0/28) | <0.001**^c^** |
| **B) 10 cm vs. 30 cm** | **10 cm** | **30 cm** |  |
| Soil pH | 5.7 (4.8-6.5) | 5.7 (5.0-6.7) | 0.001 |
| Moisture (% vsw) | 16 (9.0-23.0) | 14 (7.0-24.0) | <0.001 |
| **^b^**Conductivity (salinity) (uS/cm) | 53 (16.0-419) | 27 (9.0-216) | <0.001 |
| Total N (%) | 0.17 (0.04-0.62) | 0.03 (0-0.27) | <0.001 |
| Organic C (%) | 6.6 (3.0-15.9) | 3.9 (0-0.27) | <0.001 |
| Ratio total N/organic C (x 10e3) | 21.7 (10.2-63.4) | 17.1 (0-40.1) | <0.001 |
| Gravel (%) | 0 (0-27.5) | 0 (0-12.5) | 0.037 |
| Red/orange soil color (Y, N) | 0% (0/50) | 68% (34/50) | <0.001 |
| Clay (Y, N) | 66% (33/50) | 44% (22/50) | <0.001 |

**^a^** Generalized linear mixed models (GLMMs) used outcomes for soil variables and predictors sorted according to grid area (northern vs southern) or depth (10 vs 30cm), random effects for hole and an exponential spatial covariance structure were included (packages NLME (lme()) or MASS (glmmPQL()) in R). Model residuals were checked for lack of pattern across fitted values. Gaussian distributed errors were fitted for soil pH and moisture, binomial for bare soil, soil colour and clay and a Gamma distribution (log link) for the remaining variables (a small constant <1 was added to all values if the variable also contained zeroes).

**^b^** Two extreme values of conductivity (values >1,000 uS/cm) were excluded from the analysis.

**^c^** Due to 100% of samples in southern area consisting of bare soil, P-value is not readily defined.
